# Layer- and subregion-specific electrophysiological and morphological changes of the medial prefrontal cortex in a mouse model of neuropathic pain

**DOI:** 10.1038/s41598-019-45677-z

**Published:** 2019-07-01

**Authors:** Miodrag Mitrić, Anna Seewald, Giorgia Moschetti, Paola Sacerdote, Francesco Ferraguti, Kai K. Kummer, Michaela Kress

**Affiliations:** 1 Division of Physiology, Medical University of Innsbruck, Innsbruck, Austria; 2 Department of Pharmacology, Medical University of Innsbruck, Innsbruck, Austria; 3 Department of Pharmacological and Biomolecular Sciences, University of Milan, Milan, Italy

**Keywords:** Chronic pain, Excitability

## Abstract

Chronic neuropathic pain constitutes a serious public health problem, but the disease mechanisms are only partially understood. The involvement of different brain regions like the medial prefrontal cortex has already been established, but the comparison of the role of different subregions and layers is still inconclusive. In the current study, we performed patch-clamp recordings followed by anatomical reconstruction of pyramidal cells from different layers of the prelimbic and infralimbic subregions of the medial prefrontal cortex in neuropathic (spared nerve injury, SNI) and control mice. We found that in the prelimbic cortex, layer 2/3 pyramidal cells from SNI mice exhibited increased excitability compared to sham controls, whereas prelimbic layer 5 pyramidal neurons showed reduced excitability. Pyramidal cells in both layer 2/3 and layer 5 of the infralimbic subregion did not change their excitability, but layer 2/3 pyramidal cells displayed increased dendritic length and branching. Our findings support the view that chronic pain is associated with subregion- and layer-specific changes in the medial prefrontal cortex. They therefore provide new insights into the mechanisms underlying the chronification of pain.

## Introduction

Chronic neuropathic pain constitutes a serious public health problem that affects a large number of individuals worldwide^1–4^. The disease mechanisms underlying the chronification of pain are still not completely understood, as is its connection with frequently occurring cognitive and affective impairments^5,6^. Only a decade ago, research has started to systematically investigate pain related changes of neuronal processing at supraspinal levels, including the brainstem, as well as subcortical and cortical brain regions (for review see^7^ and^8^). The medial prefrontal cortex (mPFC), which is primarily known for its prominent role in attention and goal-directed behavior^9^, provides top-down regulation of sensory and affective processes^10^, including inhibition of both sensory and affective nociceptive signals by descending projections to various brain and spinal cord regions^11–13^. In both human subjects and rodent models, the mPFC undergoes structural as well as functional changes in chronic pain states^14–21^, which are reflected by cognitive deficits and decreased attention (for review see^22^). In line with this, attention directed to painful stimuli increases reported pain intensities^23^ and attention distraction reduces subjective pain intensities in human healthy volunteers^24,25^, thereby suggesting a strong impact of attention on the chronification of pain.

From an anatomical as well as functional perspective and based on their projection targets, the mPFC can be subdivided into the anterior cingulate cortex (ACC), the prelimbic (PrL) and the infralimbic cortex (IL)^26^. The ACC extends rostro-caudally, shows increased activity during acute pain perception as well as during persistent pain conditions^27,28^, and reduction of ACC activity has been found to relieve pain^29^. For the remaining mPFC subregions, the majority of studies have so far not separated IL and PrL contributions to functional and structural changes in pain processing^15,30–32^. Also, effects of chronic pain on neuronal populations of different layers of these regions have not yet been investigated systematically.

The current study is therefore the first to systematically dissect the effects of persistent nociceptive activity from the spared nerve injury model of neuropathic pain on pyramidal neurons of layers 2/3 and layer 5 in the PrL and IL mPFC subregions, providing evidence for subregion- and layer-specific alterations of electrophysiological properties and dendritic complexity.

## Methods

### Animals

All procedures involving animals were carried out in accordance with the Ethics Guidelines of Animal Care (Medical University of Innsbruck), as well as the European Communities Council Directive of 22 September 2010 on the protection of animals used for scientific purposes (2010/63/EU), and approved by the Austrian National Animal Experiment Ethics Committee of the Austrian Bundesministerium für Wissenschaft und Forschung (permit number BMWF-66.011/0087-WF/V/3b/2016). C57BL/6 J mice (Janvier Labs) were housed under specific pathogen-free (SPF) conditions. Animals were maintained at constant room temperature of 24 °C on a 12 h light/dark cycle with lights on from 07:00 to 19:00 and had *ad libitum* access to autoclaved pelleted food and water. A total of nineteen male adult mice (8–10 weeks old) were used for the experiments. The animals were individually housed for at least 7 days before surgery.

### Spared nerve injury (SNI)

Mice were subjected to SNI as an animal model of persistent peripheral neuropathic pain according to the method of^33^. Animals were anesthetized with a mixture of ketamine (Ketasol®, 20 mg/ml) and xylazine (Xylasol®, 2 mg/ml) in PBS (5 µl/g body weight, i.p). The sciatic nerve of the left hind leg was exposed at the level of the trifurcation into the sural, tibial, and common peroneal nerves. The tibial and common peroneal nerves were tightly ligated and transected 1–2 mm distal to the ligation, leaving the sural branch intact. Sham-operated mice without nerve transection served as controls as their sciatic nerves were exposed without additional manipulations. The successful induction of mechanical allodynia was confirmed using a dynamic plantar aesthesiometer (Ugo Basile; Supplemental Fig. 1).

### Dynamic plantar test

In order to assess hind paw mechanical sensitivity, mice were habituated to a plexi-glass chamber with a metal wire mesh floor for 15 min. The mechanical stimulus was delivered perpendicularly to the lateral side of the plantar surface of the paw (sural nerve innervation territory) by an automated testing device (Dynamic Plantar Aesthesiometer, Ugo Basile). A 0.5 mm steel rod was pushed against the hind paw with ascending force of 0 to 10 g over a period of 10 s at a rate of 1 g/s. The mechanical stimulus automatically stopped when the animal withdrew its hind paw, and the threshold was calculated as an average of three consecutive trials in both paws.

### Acute brain slice preparation

Acute coronal brain slices were prepared as previously described^34^. Briefly, 7 days after surgery the animals were anesthetized with isoflurane (Forane®, AbbVie) and decapitated. Brains were rapidly removed and immersed in ice-cold oxygenated protective artificial cerebrospinal fluid (aCSF, 95% O_2_, 5% CO_2_) containing (in mM): N-methyl-D-glucamine 110, HCl 110, KCl 2.5, NaH_2_PO_4_ 1.2, NaHCO_3_ 25, D-glucose 25, MgSO_4_ 10, CaCl_2_ 0.5, Na-ascorbate 1 and Na-pyruvate 2.9, osmolarity: ~310 mOsm/kg, pH adjusted to 7.4 with HCl^35^. The brains were trimmed with a scalpel blade and glued onto the stage of a vibrating microtome (VT1200S, Leica Microsystems). Coronal slices (thickness 300 µm) containing both the prelimbic and the infralimbic subregions of the prefrontal cortex were cut in oxygenated ice-cold protective aCSF and subsequently incubated at 32–34 °C for 5 minutes. After this recovery period, the slices were transferred to standard oxygenated aCSF containing (in mM): NaCl 125, NaHCO_3_ 25, D-glucose 25, KCl 2.5, NaH_2_PO_4_ 1.25, CaCl_2_ 2 and MgCl_2_ 1, osmolarity: ~310 mOsm/kg, pH adjusted to 7.4 with HCl^36^ at room temperature for at least 30 minutes before the electrophysiological recordings.

### Electrophysiological recordings and analysis

Sections were visualized in a recording chamber of an upright microscope (BX51WI, Olympus) equipped with differential infrared contrast optics (DIC), and continuously perfused with oxygenated standard aCSF (2–3 ml/min). Synaptic transmission was blocked using CNQX (6-cyano-7-nitroquinoxaline-2,3-dione, 5 μM), picrotoxin (5 μM) and D-AP5 (5 μM). Patch pipettes were pulled from borosilicate glass capillaries (Science Products) using a flaming micropipette puller (P97, Sutter Instruments) resulting in a pipette resistance of 3–6 MΩ after filling. The pipette solution for current-clamp recordings contained (in mM): 135 K-gluconate, 20 KCl, 2 MgCl_2_, 10 HEPES, 0.1 EGTA, 2 Mg-ATP, 0.3 Na-GTP and 3–5 mg/ml biocytin (osmolarity: ~295 mOsm/kg, pH adjusted to 7.3 with KOH). Recordings were performed at room temperature with an EPC 10 amplifier and PatchMaster software v2x73.1 (HEKA). Data were filtered at 2.9 kHz using a Bessel filter, and the sampling rate ranged from 20 to 50 kHz depending on the protocol applied. Access resistance (R_s_) was monitored in the voltage-clamp configuration by analysing capacitive transients during 10 ms square wave depolarizing pulses. Recordings were included only when a GΩ seal formed prior to whole-cell access with a R_s_ of less than 20 MΩ.

Whole-cell patch clamp recordings were obtained from pyramidal cells in layers 2/3 and layer 5 depending on their perpendicular distance from the midline, 100–300 µm and 300–500 µm, respectively. One coronal slice was used per animal and chosen according to the coordinates from^37^, relative to Bregma, in mm: AP +1.70. Prelimbic and infralimbic subregions were targeted based on their vertical distance from the dorsal end of the midline, 600–1300 µm and 1450–1900 µm, respectively. Data acquisition started 10 min after entering the whole-cell mode allowing sufficient stabilization of the recording. Pyramidal neurons from different layers and subregions were recorded in each of the slices and were distinguished from interneurons by their shape, spiking pattern and action potential width^38^. In addition, their identity was confirmed by their large apical dendrites, which were visualized through immunohistochemical processing.

None of the neurons showed spontaneous activity, and all parameters were obtained at resting membrane potential (RMP) that was determined by averaging a 1 min recording period at 0 pA in current clamp mode (Supplemental Fig. 2; Supplemental Table 1). Depolarizing current steps (50 ms) with an increment of 10 pA were applied every 3 s via the recording electrode until an action potential (AP) was elicited. Action potential threshold current (I_AP_) was defined as the minimum amount of current needed to induce the first AP. AP analysis was performed as described previously^39^. Briefly, five consecutive APs induced by a 50 ms I_AP_ +10 pA depolarizing current were averaged and analyzed using the FitMaster software (HEKA). AP amplitude, afterhyperpolarization (AHP) and AHP time to peak (t_AHP_) were determined relative to the AP threshold. AP duration was determined at its half-amplitude as AP half-width. The first derivative was used to extract the maximum speed of depolarization (dv/dt_max_) and repolarization (dv/dt_min_). AP threshold voltage was taken at the point where the depolarization speed first exceeded 10 mV/ms.

Additional biophysical parameters were extracted from voltage responses to 500 ms current pulses at 0.2 Hz ranging from −100 to +500 pA in 20 pA increments. Input resistance (R_in_) was obtained by linear fit of the I/V curve from −100 to 0 pA. Membrane time constant (τ_m_) and membrane capacitance (C_m_) were obtained by an exponential fit to the voltage response following a −40 mV hyperpolarizing step current^40^. AP latency of the 1^st^ AP was measured as time from current onset to crossing the voltage threshold of 0 mV. Input-Frequency (I-F) slope was calculated as linear coefficient of the 2^nd^ order polynomial fit of the AP frequency vs. current injection relation. Mean inter-spike interval (ISI) and adaptation ratio (1^st^ ISI/9^th^ ISI) were taken from the first trace with at least 10 APs. Sag ratio (%) was calculated from the current injection that would cause a hyperpolarization of approximately −7.5 mV^38^ as (V_ss_ − V_min_)/(V_min_ − V_rmp_), with V_min_ being the minimum value reached after the beginning of the current injection, V_ss_ being the voltage at steady-state and V_rmp_ being the resting membrane potential. After experiments were completed, slices were immersed in a 4% paraformaldehyde (PFA) fixative for at least 24 h prior to subsequent visualization of the filled neurons.

### Biocytin visualization

Neuronal morphology was assessed using a diaminobenzidine (DAB; Sigma-Aldrich) staining protocol as follows: slices were washed three times in Tris-buffered saline (TBS; 0.9% NaCl, 0.05 M Tris, pH 7.4) for 10 min each. Subsequently, slices were left for overnight incubation in a 1:100 solution of avidin-biotinylated horseradish peroxidase (ABC-Elite) in 1% Bovine Serum Albumin (BSA)-TBS at 4 °C. On the next day, sections were washed in TBS and Tris buffer (TB; 0.05 M Tris, pH 7.4), before incubating them in 0.5 mg/ml DAB in TB with Nickel (4 mg/ml). Hydrogen peroxide (0.003%) was added to TB in order to start the peroxidase reaction. Sections were then rinsed with TBS three times and mounted on gelatin-coated slides, and left to air-dry. Finally, they were dehydrated and coverslipped using Eukitt (Marienfeld Lab. Glassware, Germany).

### 3D reconstruction and Sholl analysis

Biocytin-stained neurons were 3D reconstructed using the NEUROLUCIDA® software (MBF Bioscience). All cells included in the analysis were checked for optimal filling in both apical and basal dendrites. Before tracing, cells were visually inspected and discarded if proximal branches of the dendrites appeared cut. Somata, dendrites and axonal branches were drawn with a 1.3 NA 100x oil objective lens (Olympus BX51). Morphological parameters were analyzed with NEUROEXPLORER® software (MBF Bioscience), including complexity, somatic area, total dendritic length and branching of the dendrites. The area of the cell body was calculated by referring to the boundary of the cell body within a 2D area. To analyze the branch order, the centrifugal ordering system was used as it provides information about both the topological distance as well the amount of branching within a tree. For each neuron, tracing was performed by following the dendrites from the soma to the periphery. Total dendritic length was calculated as the sum of the length of all the branches within a dendritic tree, whereas the mean length was obtained by dividing the total length by the number of primary branches. The complexity was calculated as follows: Complexity = (sum of the terminal orders + number of terminals) * (total dendritic length/number of primary dendrites).

In order to determine dendritic complexity in more detail, Sholl analysis was performed using the NEUROEXPLORER® software. Concentric Sholl rings were set at 10 µm intervals from the soma. The analysis was based on calculating the number of intersections and dendritic length per Sholl ring interval.

### Statistical analysis

For statistical analyses, GraphPad Prism 7 (two-tailed Student’s t-test, 2-way ANOVA followed by Sidak’s multiple comparisons test) and Origin 9 (polynomial curve fitting), were used as applicable. The level of statistical significance was predefined at p < 0.05.

## Results

As a first step, we confirmed SNI induced mechanical hypersensitivity in the operated paw by measuring mechanical withdrawal thresholds at baseline and 7 days after surgery. As expected, SNI operated mice exhibited a significant reduction in the paw withdrawal threshold of the operated paw compared to sham controls as well as compared to baseline measurements (2-way RM ANOVA, p < 0.001; Supplemental Fig. 1). In addition, the withdrawal threshold of the non-operated (contralateral) paw was unaltered (2-way RM ANOVA, p > 0.05; Supplemental Fig. 1).

In order to investigate whether the spared nerve injury (SNI) mouse model induces layer specific and subregion specific electrophysiological and morphological changes in pyramidal cells of the medial prefrontal cortex (mPFC), we performed whole-cell patch clamp recordings and subsequent morphological analyses in a subset of recorded neurons 7 days after surgery. The prelimbic (PrL) cortex is set along the midline and is bordered dorsally by the anterior cingulate cortex and ventrally by the infralimbic (IL) cortex. In this study we investigated layer 2/3 and layer 5 pyramidal cells of both PrL and IL cortical areas.

Recordings were performed in the presence of blockers of fast synaptic transmission (5 µM CNQX, 5 µM D-AP5, 5 µM picrotoxin) and were included in the analysis only if the resting membrane potential was stable and below −60 mV, and if pyramidal cell like morphology was confirmed. Experimenters were blind to the treatment condition.

### SNI increases the excitability of layer 2/3 pyramidal cells of the prelimbic (PrL) cortex

**Figure 1:**
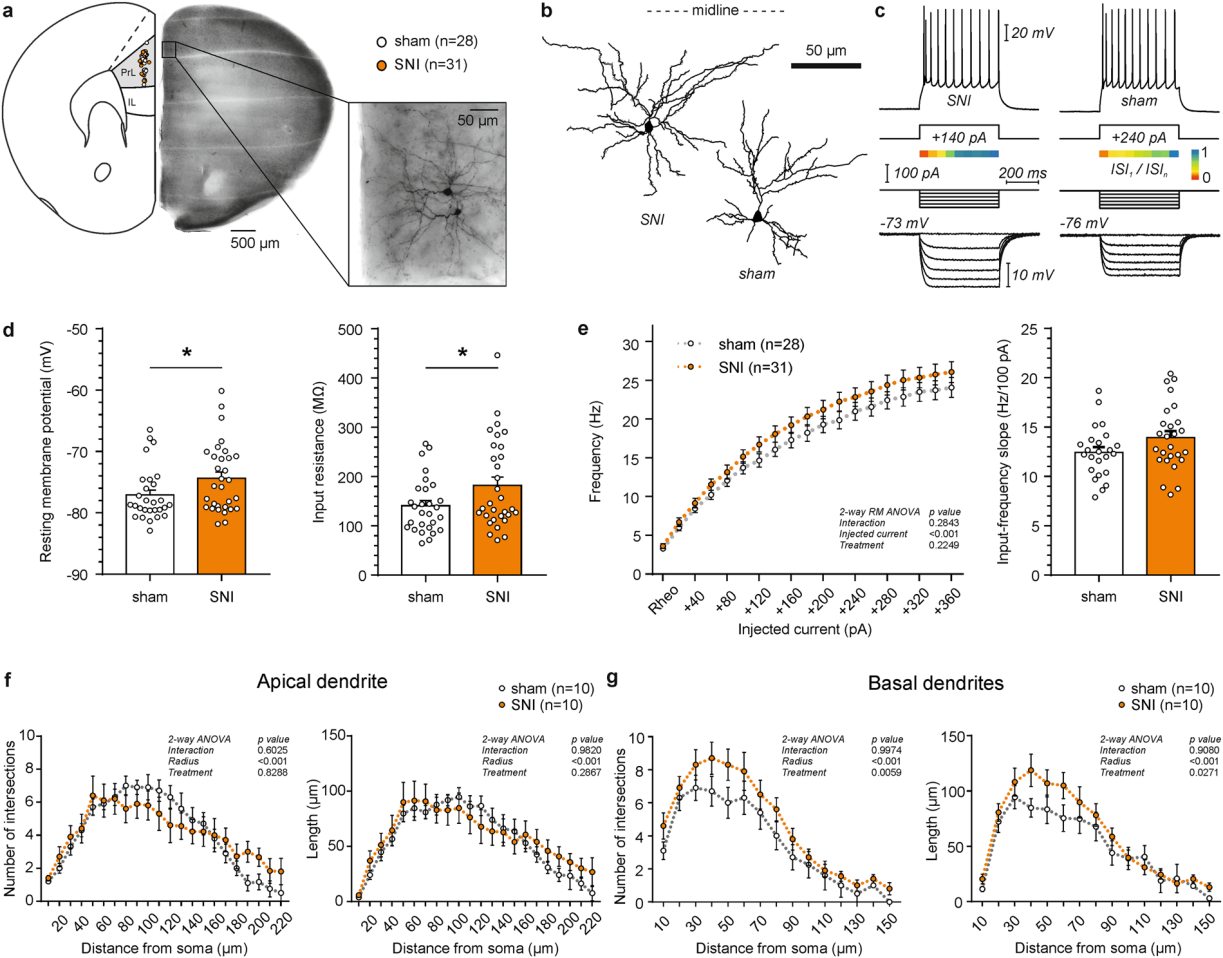
Firing rate and Sholl analysis of layer 2/3 pyramidal cells in the prelimbic (PrL) cortex of SNI and sham mice. (**a**) Location of recorded neurons in the hemisphere contralateral to the injured paw. Recordings were performed in the presence of blockers of fast synaptic transmission (5 µM CNQX, 5 µM D-AP5 and 5 µM picrotoxin). Inset shows DAB stained, biocytin filled neurons in the targeted region. (**b**) Representative examples of reconstructed L2/3 PrL pyramidal cells. (**c**) Representative electrophysiological profile of L2/3 PrL pyramidal neurons in the SNI (left) and sham (right) group. Top, firing pattern when a minimum of 10 action potentials was elicited in response to the corresponding current step. Below, adaptation ratios from 1^st^ to the n-1^st^ interspike interval relative to the last (n^th^) presented as a heat map. Bottom, response to hyperpolarizing current injections that would be used to calculate the input resistance and the voltage sag ratio. (**d**) Column graphs showing the resting membrane potential and input resistance of the two groups (*p < 0.05, Student’s t-test). (**e**) Firing rate as a function of injected current in SNI mice compared to sham controls (p > 0.05, 2-way ANOVA with repeated measures). Input-frequency slope calculated as a linear coefficient of the polynomial fitting of the curves (p > 0.05, Student’s t-test). (**f**) Sholl analysis of the reconstructed apical dendrites in SNI and sham treated mice (p > 0.05, 2-way ANOVA). (**g**) Sholl analysis of the basal dendrites shows an increase in the number of intersections and length per distance from soma in SNI mice (p < 0.05, 2-way ANOVA).

In order to investigate the main input region of the prelimbic (PrL) prefrontal cortex, we first targeted PrL layer 2/3 (L2/3) pyramidal cells of sham and SNI treated mice by mapping the landmarks of the coronal slices to the mouse brain stereotaxic atlas (^37^; Fig. 1a). By measuring the distance from the dorsal end of the midline, we ensured the same localization of recorded neurons between the two treatment groups (Table 1; x/y coordinates: sham 917.64 ± 28.98/222.14 ± 5.27 µm vs. SNI 912.33 ± 24.81/230.87 ± 6.89 µm; Student’s t-test, p_x_ = 0.89/p_y_ = 0.33). Whole-cell recordings showed that passive membrane properties of PrL L2/3 pyramidal neurons differed between SNI and sham-operated mice (Fig. 1d; Table 1). By measuring the resting membrane potential, we observed a more depolarized state of L2/3 pyramidal neurons after SNI, but not sham treatment (SNI −74.37 ± 0.99 mV vs. sham −77.08 ± 0.80 mV; Student’s t test, p = 0.04). Neurons from SNI mice showed a higher input resistance (SNI: 182.44 ± 16.07 MΩ; sham: 140.96 ± 10.32 MΩ) compared to the sham group (Student’s t-test, p = 0.04). The membrane time constant (τ_m_) was also significantly higher in SNI mice compared to sham (SNI 29.14 ± 2.52 ms vs. sham 22.33 ± 1.56 ms; Student’s t-test, p = 0.03). Correspondingly, we observed a trend towards increased firing rate in response to 500 ms depolarizing current injection in the SNI group (Fig. 1e; Table 1; I-F slope (pA/100 Hz): SNI 13.94 ± 0.64 vs. sham 12.45 ± 0.52; Student’s t-test, p = 0.08). AP properties were similar for both groups (Table 1). The firing pattern in both groups exhibited strong adaptation as indicated by the ratio of the first and last interspike interval obtained from the first trace with at least 10 APs (ISI_1_/ISI_n_: SNI 0.33 ± 0.02 vs. sham 0.31 ± 0.02; Student’s t-test, p > 0.05). The voltage sag ratio indicative for the activation of hyperpolarization-activated cyclic nucleotide–gated (HCN) channels in response to a hyperpolarizing current injection of ~−7.5 mV was similar in both groups (sham: 4.55 ± 0.25%; SNI: 4.27 ± 0.27%, Student’s t-test, p > 0.05) and consistent with low HCN channel expression in L2/3 pyramidal cells compared to deeper cortical layers^38^.

**Table 1:** Electrophysiological and morphological comparison of L2/3 prelimbic pyramidal cells between SNI and sham treated mice.

Electrophysiology	sham (n = 28)	SNI (n = 31)	P value	Morphology	sham (n = 10)	SNI (n = 10)	P value
**RMP (mV)**	−77.08 ± 0.81	−74.37 ± 1.01	*0.0430	**Cell body**			
**Input resistance (MΩ)**	140.96 ± 10.51	182.44 ± 16.34	*0.0415	x (µm)	917.64 ± 29.51	912.33 ± 25.24	0.8911
**Membrane capacitance (pF)**	171.89 ± 10.41	165.37 ± 8.26	0.6222	y (µm)	222.14 ± 5.37	230.87 ± 7.01	0.3322
**τ**_**m**_ **(ms)**	22.33 ± 1.59	29.14 ± 2.56	*0.0316	Area (µm²)	120.98 ± 10.17	102.10 ± 6.56	0.1361
**Sag ratio (%)**	4.55 ± 0.26	4.27 ± 0.28	0.4753	**Basal dendrites**			
**Current threshold (pA)**	214.29 ± 16.50	176.77 ± 15.67	0.1048	Number	3.80 ± 0.42	5.00 ± 0.47	0.0725
**Voltage threshold (mV)**	−39.06 ± 0.38	−40.09 ± 0.60	0.1656	Nodes	7.10 ± 0.99	7.00 ± 0.67	0.9343
**AP amplitude (mV)**	57.14 ± 0.54	58.12 ± 0.48	0.1803	Ends	11.20 ± 1.24	12.10 ± 1.06	0.5872
**dv/dt**_**max**_ **(mV/ms)**	430.38 ± 19.01	459.79 ± 17.55	0.2597	Total length (µm)	672.96 ± 94.98	899.66 ± 104.99	0.1267
**dv/dt**_**min**_ **(mV/ms)**	−56.38 ± 1.87	−60.39 ± 1.86	0.1350	Mean length (µm)	183.02 ± 21.14	181.54 ± 18.09	0.9584
**AHP (mV)**	−46.89 ± 0.51	−47.82 ± 0.42	0.1617	Complexity	6813.75 ± 1629.88	6031.35 ± 919.15	0.6808
**AHP time to peak (ms)**	4.99 ± 0.14	4.98 ± 0.13	0.9691	**Apical dendrite**			
**50% AP width (ms)**	1.33 ± 0.04	1.27 ± 0.04	0.2444	Nodes	12.00 ± 1.26	11.10 ± 2.13	0.7198
**I-F slope (Hz/100pA)**	12.45 ± 0.53	13.94 ± 0.66	0.0859	Ends	13.00 ± 1.26	12.30 ± 2.17	0.7833
**1** ^**st**^ **AP latency (ms)**	167.99 ± 8.30	179.67 ± 15.78	0.5279	Total length (µm)	1199.76 ± 82.07	1212.78 ± 226.05	0.9574
**Average ISI (ms)**	51.11 ± 1.20	48.85 ± 0.43	0.0612	Complexity	88582.7 ± 415723.45	114749.12 ± 43933.76	0.5819
**Ratio ISI**_**1**_ **/ISI**_**n**_	0.31 ± 0.02	0.33 ± 0.02	0.5513				

Values are mean ± SEM, with sample size in parenthesis. P-values were determined by Student’s t-test. x, y cell coordinates relative to the dorsal apex and midline of the coronal slice respectively, RMP resting membrane potential, τ_m_ membrane time constant, AP action potential, AHP after-hyperpolarization, dv/dt_max_ peak depolarization velocity, dv/dt_min_ min peak repolarization velocity, ISI interspike interval, I-F input-frequency; *p < 0.05.

Anatomical reconstruction of 10 recorded pyramidal neurons per group showed no apparent differences in the length or complexity of basal and apical dendrites (Table 1). Furthermore, consistent with the unaltered membrane capacitance (SNI 165.37 ± 8.12 pF vs. sham 171.89 ± 10.22 pF; Student’s t test, p > 0.05), there was no difference in the surface area of the somata between the two groups (SNI 102.10 ± 6.23 µm^2^ vs. sham 120.98 ± 9.65 µm^2^; Student’s t-test, p > 0.05). However, more detailed Sholl analysis^41^ revealed that basal dendrites of L2/3 pyramidal neurons from SNI treated mice had a higher number of intersections (Fig. 1g; 2-way ANOVA, treatment effect, p = 0.0059) and an increased length per distance compared to sham animals (Fig. 1g; 2-way ANOVA, treatment effect, p = 0.027). In contrast, in the apical dendrites the number of intersections and the dendritic length was similar between the two groups of mice (Fig. 1f; 2-way ANOVA, treatment effect, p > 0.05). Taken together, these findings suggest that neuropathic pain leads to a fine structural remodeling of the basal dendritic arbors of PrL L2/3 pyramidal neurons.

### SNI reduces the firing rate of prelimbic layer 5 pyramidal neurons

**Figure 2:**
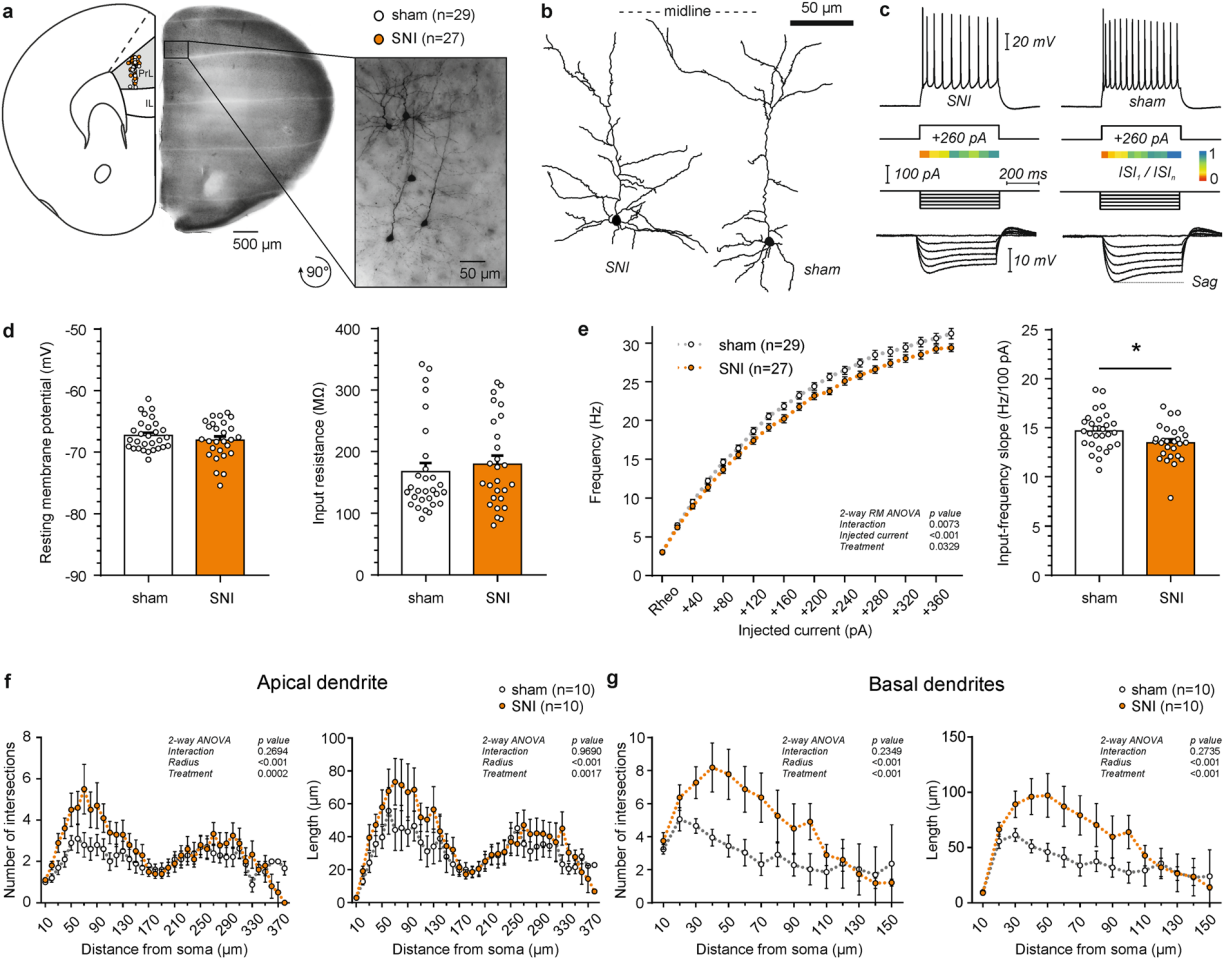
Firing rate and Sholl analysis of layer 5 pyramidal cells in the prelimbic (PrL) cortex of SNI and sham mice. (**a**) Location of recorded neurons in the hemisphere contralateral to the injured paw. Recordings were performed in the presence of blockers of fast synaptic transmission (5 µM CNQX, 5 µM D-AP5 and 5 µM picrotoxin). Inset shows DAB stained, biocytin filled neurons in the targeted region. (**b**) Representative examples of reconstructed L5 PrL pyramidal cells. (**c**) Representative electrophysiological profile of L5 PrL pyramidal neurons in the SNI (left) and sham (right) group. Top, firing pattern when a minimum of 10 action potentials was elicited in response to the corresponding current step. Below, adaptation ratios from 1^st^ to the n-1^st^ interspike interval relative to the last (n^th^) presented as a heat map. Bottom, response to hyperpolarizing current injections to calculate input resistance and voltage sag ratio. (**d**) Bar graphs of the resting membrane potential and the input resistance of the two groups (p > 0.05, Student’s t-test). (**e**) Firing rate as a function of injected current showing a reduced discharge in SNI mice compared to sham controls (p < 0.05, 2-way ANOVA with repeated measures). Input-frequency slope calculated as a linear coefficient of the polynomial fitting of the curves (*p < 0.05, Student’s t-test). (**f**) Sholl analysis of reconstructed apical dendrites shows an increase in the number of intersections and length per distance from soma in SNI mice (p < 0.05, 2-way ANOVA). (**g**) Sholl analysis of the basal dendrites shows an increase in the number of intersections and length per distance from soma in SNI mice (p < 0.05, 2-way ANOVA).

We next tested if pyramidal neurons in L5, the main output layer of the neocortex, were altered in SNI treated mice 7 days after surgery. In recordings from PrL L5 pyramidal cells at the same relative coordinates in sham and SNI mice (Table 2; x/y coordinates: sham 933.61 ± 25.52/427.55 ± 6.86 µm vs. SNI 888.11 ± 25.85/425.96 ± 10.04 µm; Student’s t-test, p_x_ = 0.22/p_y_ = 0.90) we found no difference in passive membrane properties between the two treatment groups (Table 2; Fig. 2d). The resting membrane potential was −67.29 ± 0.45 mV for sham and −68.06 ± 0.58 mV for SNI (Student’s t-test, p = 0.31). Correspondingly, input resistance (SNI 179.50 ± 13.61 MΩ vs. sham 167.62 ± 13.52 MΩ; Student’s t-test, p = 0.55) and AP parameters were similar in both groups (Table 2). Lower firing rates were detected in neurons of the SNI group in response to 500 ms depolarizing current injections (Fig. 2e; repeated measures 2-way ANOVA, treatment effect, p = 0.033). Consistently, the I-F linear slope was reduced in the SNI group following a polynomial fitting of the I-F relationship (SNI 13.47 ± 0.35 Hz/100 pA vs. sham 14.69 ± 0.35 Hz/100 pA; Student’s t test, p = 0.02). Overall, PrL L5 pyramidal neurons exhibited a prominent and similar voltage sag of 16.47 ± 1.60% in sham and 14.99 ± 1.76% in SNI.

**Table 2:** Electrophysiological and morphological comparison of L5 prelimbic pyramidal cells between SNI and sham treated mice.

Electrophysiology	sham (n = 29)	SNI (n = 27)	P value	Morphology	sham (n = 10)	SNI (n = 10)	P value
**RMP (mV)**	−67.29 ± 0.45	−68.06 ± 0.60	0.3072	**Cell body**			
**Input resistance (MΩ)**	167.62 ± 13.76	179.50 ± 13.87	0.5464	x (µm)	933.61 ± 25.97	888.11 ± 26.34	0.2244
**Membrane capacitance (pF)**	193.56 ± 5.82	195.37 ± 6.25	0.8323	y (µm)	427.55 ± 6.98	425.96 ± 10.23	0.8972
**τ**_**m**_ **(ms)**	38.59 ± 2.71	42.51 ± 3.01	0.3350	Area (µm²)	131.81 ± 6.46	145.80 ± 7.61	0.1781
**Sag ratio (%)**	16.47 ± 1.63	14.99 ± 1.79	0.5431	**Basal dendrites**			
**Current threshold (pA)**	114.83 ± 5.41	117.70 ± 4.95	0.6975	Number	3.60 ± 0.34	5.00 ± 0.39	*0.015
**Voltage threshold (mV)**	−43.50 ± 0.52	−43.03 ± 0.57	0.5471	Nodes	3.70 ± 0.79	6.50 ± 1.77	0.1660
**AP amplitude (mV)**	57.84 ± 0.60	57.44 ± 0.64	0.6507	Ends	7.30 ± 0.92	11.60 ± 2.14	0.0809
**dv/dt**_**max**_ **(mV/ms)**	467.14 ± 16.17	471.62 ± 19.55	0.8599	Total length (µm)	463.68 ± 115.42	805.40 ± 163.92	0.1055
**dv/dt**_**min**_ **(mV/ms)**	−63.21 ± 1.21	−64.65 ± 1.45	0.4471	Mean length (µm)	127.78 ± 27.35	150.67 ± 19.54	0.5045
**AHP (mV)**	−48.88 ± 0.47	−48.56 ± 0.42	0.6178	Complexity	2880.24 ± 1455.72	5802.88 ± 2259.73	0.2913
**AHP time to peak (ms)**	5.04 ± 0.22	4.84 ± 0.17	0.4723	**Apical dendrite**			
**50% AP width (ms)**	1.22 ± 0.02	1.18 ± 0.03	0.3547	Nodes	10.90 ± 1.62	12.70 ± 1.71	0.4545
**I-F slope (Hz/100pA)**	14.69 ± 0.35	13.47 ± 0.36	*0.0191	Ends	11.90 ± 1.62	13.90 ± 1.70	0.4063
**1** ^**st**^ **AP latency (ms)**	160.47 ± 11.41	173.42 ± 14.77	0.4872	Total length (µm)	1077.19 ± 122.03	1325.89 ± 160.00	0.2324
**Average ISI (ms)**	50.37 ± 0.25	50.51 ± 0.29	0.7206	Complexity	100610.14 ± 22502.23	152935.04 ± 44373.99	0.3069
**Ratio ISI**_**1**_ **/ISI**_**n**_	0.39 ± 0.03	0.33 ± 0.03	0.1618				

Values are mean ± SEM, with sample size in parenthesis. P-values were determined by Student’s t test. x, y cell coordinates relative to the dorsal apex and midline of the coronal slice respectively, RMP resting membrane potential, τ_m_ membrane time constant, AP action potential, AHP after-hyperpolarization, dv/dt_max_ peak depolarization velocity, dv/dt_min_ min peak repolarization velocity, ISI interspike interval, I-F input-frequency; *p < 0.05.

Even though none of the primary morphological parameters of the dendritic tree of L5 pyramidal cells differed between SNI and sham animals (Table 1), Sholl analysis revealed a significant increase in both apical and basal dendrites in the length per distance (2-way ANOVA, treatment effect: apical dendrites, p < 0.002; basal dendrites, p < 0.001) as well as number of intersections (2-way ANOVA, treatment effect: apical dendrites, p < 0.001; basal dendrites, p < 0.001) in SNI mice (Fig. 2f,g).

### SNI extends the dendritic arbor of infralimbic layer 2/3 pyramidal cells without altering their biophysical properties

**Figure 3:**
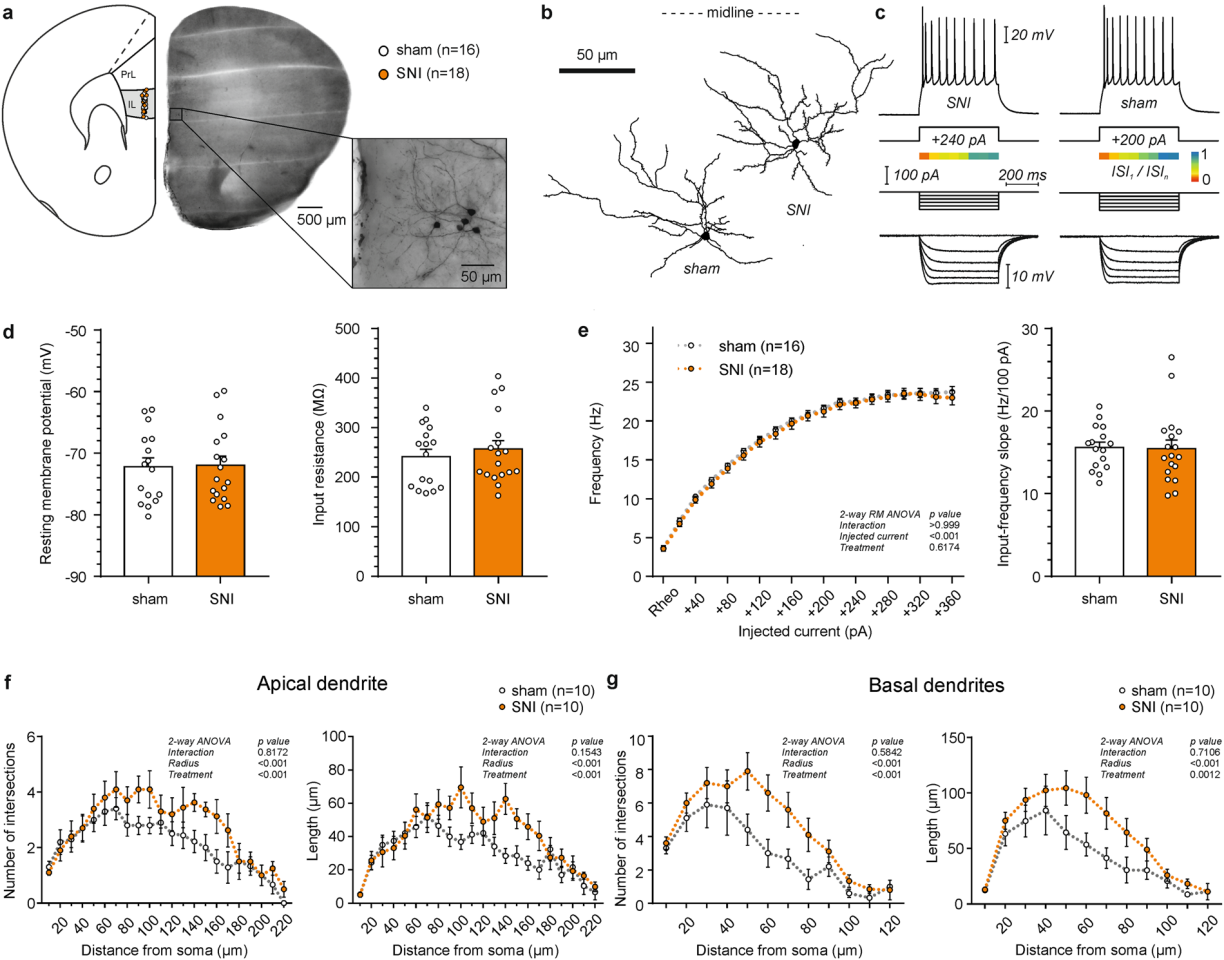
Firing rate and Sholl analysis of layer 2/3 pyramidal cells in the infralimbic (IL) cortex of SNI and sham mice. (**a**) Location of recorded neurons in the hemisphere contralateral to the injured paw. Recordings were performed in the presence of blockers of fast synaptic transmission (5 µM CNQX, 5 µM D-AP5 and 5 µM picrotoxin). Inset shows DAB stained, biocytin filled neurons in the targeted region. (**b**) Representative examples of L2/3 IL pyramidal cells. (**c**) Representative electrophysiological profile of L2/3 IL pyramidal neurons in the SNI (left) and sham (right) group. Top, firing pattern when a minimum of 10 action potentials was elicited in response to the corresponding current step. Underneath, adaptation ratios from 1^st^ to the n-1^st^ interspike interval relative to the last (n^th^) presented as a heat map. Bottom, response to hyperpolarizing current injections to calculate the input resistance and the voltage sag ratio. (**d**) Column graphs showing the resting membrane potential and input resistance of the two groups (p > 0.05, Student’s t-test). (**e**) Firing rate as a function of injected current in SNI mice compared to sham controls (p > 0.05, 2-way ANOVA with repeated measures). Input-frequency slope calculated as a linear coefficient of the polynomial fitting of the curves (p > 0.05, Student’s t-test). (**f**) Sholl analysis of the reconstructed apical dendrites shows an increase in the number of intersections and length per distance from soma in SNI mice (p < 0.05, 2-way ANOVA). (**g**) Sholl analysis of basal dendrites shows an increase in the number of intersections and length per distance from soma in SNI mice (p < 0.05, 2-way ANOVA).

In agreement with previous studies in mouse models of neuropathy our data indicate that the PrL cortex undergoes functional and morphological changes already 7 days after surgery^42,43^. However, we observed that the two subregions of the mPFC, namely the PrL and the IL, were robustly different in terms of their physiological parameters in the sham group, with the IL neurons of both L2/3 and L5 showing higher excitability as well as lower membrane capacitance (Supplemental Table 2). We recorded from L2/3 pyramidal neurons of the IL cortex that were equally distributed between the treatment groups (Table 3; x/y coordinates: sham 1679.26 ± 24.19/212.53 ± 5.32 µm vs. SNI 1646.73 ± 25.83/219.83 ± 5.53 µm; Student’s t-test, p_x_ = 0.39/p_y_ = 0.37). In overt contrast to the PrL, both passive and active membrane properties of neurons were similar in SNI and control mice (Fig. 3d,e; Table 3). Conversely, morphological analysis revealed striking differences of IL L2/3 pyramidal cells between the two groups. Apical dendrites in SNI mice were significantly longer (Table 3; SNI: 802.39 ± 68.37 µm vs. sham: 592.80 ± 60.66 µm; Student’s t-test, p = 0.04) and showed higher complexity (Table 3; SNI: 39338.6 ± 6253.1 vs. sham: 19301.0 ± 3938.2; Student’s t-test, p = 0.02). For the basal dendrites in the SNI group only a trend towards an increased length (SNI: 180.67 ± 30.39 µm vs. sham: 109.24 ± 12.84 µm; Student’s t-test, p = 0.055) and complexity (SNI: 9103.0 ± 2798.1 vs. sham: 3491.2 ± 1109.4; Student’s t-test, p = 0.09) was observed. Consistently, Sholl analysis revealed a higher number of intersections (2-way ANOVA, treatment effect: apical dendrites, p < 0.001; basal dendrites, p < 0.001) and increased length (2-way ANOVA, treatment effect: apical dendrites, p < 0.001; basal dendrites, p = 0.002) of both the apical and basal dendrites compared to sham (Fig. 3f,g).

**Table 3:** Electrophysiological and morphological comparison of L2/3 infralimbic pyramidal cells between SNI and sham treated mice.

Electrophysiology	sham (n = 16)	SNI (n = 18)	P value	Morphology	sham (n = 10)	SNI (n = 10)	P value
**RMP (mV)**	−72.33 ± 1.44	−72.08 ± 1.47	0.9030	**Cell body**			
**Input resistance (MΩ)**	241.38 ± 15.16	256.81 ± 16.94	0.5066	x (µm)	1679.26 ± 25.04	1646.73 ± 26.58	0.3865
**Membrane capacitance (pF)**	123.00 ± 5.10	130.42 ± 5.91	0.3543	y (µm)	212.53 ± 5.50	219.83 ± 5.69	0.3693
**τ**_**m**_ **(ms)**	42.79 ± 4.01	44.96 ± 2.59	0.6452	Area (µm²)	100.06 ± 11.42	102.13 ± 5.81	0.8736
**Sag ratio (%)**	3.92 ± 1.51	3.57 ± 1.27	0.8611	**Basal dendrites**			
**Current threshold (pA)**	126.88 ± 9.95	119.17 ± 9.41	0.5775	Number	4.00 ± ± 0.37	4.40 ± 0.31	0.4118
**Voltage threshold (mV)**	−37.59 ± 0.61	−38.47 ± 0.66	0.3373	Nodes	5.60 ± 1.39	8.00 ± 1.42	0.2434
**AP amplitude (mV)**	57.32 ± 0.71	56.95 ± 0.85	0.7452	Ends	9.70 ± 1.71	12.60 ± 1.21	0.1829
**dv/dt**_**max**_ **(mV/ms)**	389.33 ± 17.72	412.03 ± 27.30	0.5023	Total length (µm)	464.57 ± 94.10	734.41 ± 97.31	0.0616
**dv/dt**_**min**_ **(mV/ms)**	−58.53 ± 1.98	−58.73 ± 2.73	0.9538	Mean length (µm)	109.24 ± 13.54	180.67 ± 32.03	0.0548
**AHP (mV)**	−48.28 ± 0.60	−47.51 ± 0.65	0.3962	Complexity	3491.24 ± 1169.46	9103.06 ± 2949.47	0.0939
**AHP time to peak (ms)**	5.12 ± 0.13	5.11 ± 0.18	0.9915	**Apical dendrite**			
**50% AP width (ms)**	1.28 ± 0.03	1.30 ± 0.05	0.7528	Nodes	5.60 ± 0.70	8.60 ± 0.65	*0.0058
**I-F slope (Hz/100pA)**	15.58 ± 0.66	15.46 ± 1.04	0.9248	Ends	6.60 ± 0.70	9.60 ± 0.65	*0.0058
**1** ^**st**^ **AP latency (ms)**	223.30 ± 25.92	208.74 ± 19.90	0.6550	Total length (µm)	592.80 ± 63.95	802.39 ± 72.07	*0.0432
**Average ISI (ms)**	50.46 ± 0.44	50.88 ± 0.45	0.5104	Complexity	19301.08 ± 4151.24	39338.68 ± 6591.43	*0.0192
**Ratio ISI**_**1**_ **/ISI**_**n**_	0.37 ± 0.02	0.32 ± 0.02	0.0961				

Values are mean ± SEM, with sample size in parenthesis. P-values were determined by Student’s t-test. x, y cell coordinates relative to the dorsal apex and midline of the coronal slice respectively, RMP resting membrane potential, τ_m_ membrane time constant, AP action potential, AHP after-hyperpolarization, dv/dt_max_ peak depolarization velocity, dv/dt_min_ min peak repolarization velocity, ISI interspike interval, I-F input-frequency; *p < 0.05.

### SNI does not affect the morphological and physiological features of infralimbic layer 5 pyramidal cells

**Figure 4:**
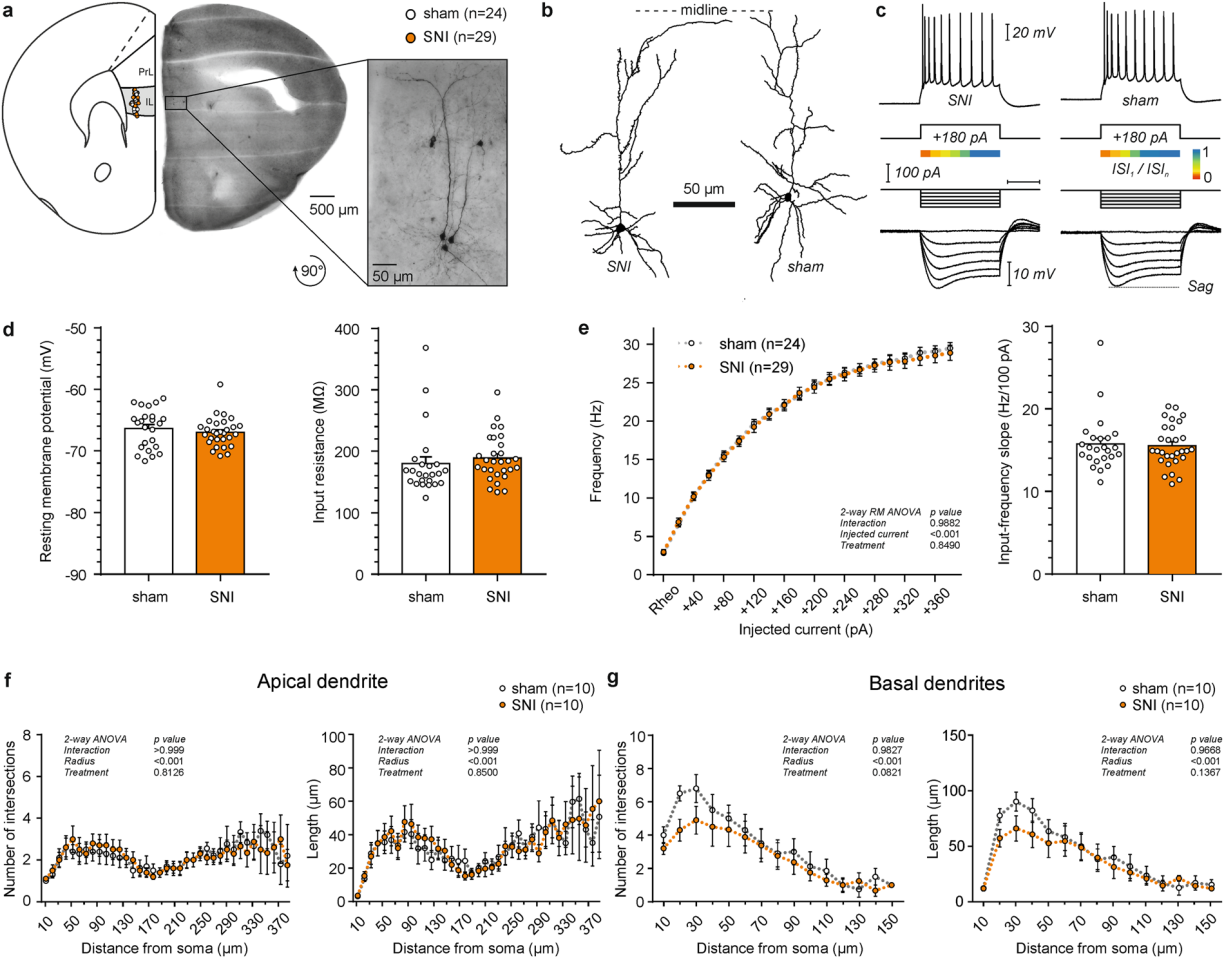
Firing rate and Sholl analysis of layer 5 pyramidal cells in the infralimbic (IL) cortex of SNI and sham mice. (**a**) Location of recorded neurons in the hemisphere contralateral to the injured paw. Recordings were performed in the presence of blockers of fast synaptic transmission (5 µM CNQX, 5 µM D-AP5 and 5 µM picrotoxin). Inset shows DAB stained, biocytin filled neurons in the targeted region. (**b**) Representative examples of L5 IL pyramidal cells. (**c**) Representative electrophysiological profile of L5 IL pyramidal neurons in the SNI (left) and sham (right) group. Top, firing pattern when a minimum of 10 action potentials was elicited in response to the corresponding current step. Below, adaptation ratios from 1^st^ to the n-1^st^ interspike interval relative to the last (n^th^) presented as a heat map. Bottom, response to hyperpolarizing current injections to calculate input resistance and voltage sag ratio. (**d**) Column graphs showing the resting membrane potential and input resistance of the two groups (p > 0.05, Student’s t-test). (**e**) Comparable firing rate as a function of injected current between the two groups (p > 0.05, 2-way ANOVA with repeated measures). Input-frequency slope calculated as a linear coefficient of the polynomial fitting of the curves (p > 0.05, Student’s t-test). (**f**,**g**) Sholl analysis of the reconstructed apical and basal dendrites in SNI and sham treated mice (p > 0.05, 2-way ANOVA).

Finally, we tested L5 pyramidal cells of the IL cortex (Table 4; x/y coordinates: sham 1679.26 ± 24.19/212.53 ± 5.32 µm vs. SNI 1646.73 ± 25.83/219.83 ± 5.53 µm; Student’s t-test, p_x_ = 0.39/p_y_ = 0.37). Resting membrane potential (SNI: −66.91 ± 0.63 mV vs. sham: −66.31 ± 0.64 mV; Student’s t-test, p = 0.44) and input resistance (sham: 179.77 ± 10.98 MΩ vs. SNI: 188.70 ± 6.92 MΩ; Student’s t-test, p = 0.49) were similar in both groups (Fig. 4d). SNI surgery did not alter the AP firing rate (Fig. 4e; repeated measures 2-way ANOVA, p > 0.05) or any of the additional AP parameters (Table 4).

**Table 4:** Electrophysiological and morphological comparison of L5 infralimbic pyramidal cells between SNI and sham treated mice.

Electrophysiology	sham (n = 24)	SNI (n = 29)	P value	Morphology	sham (n = 10)	SNI (n = 10)	P value
RMP (mV)	−66.31 ± 0.65	−66.91 ± 0.44	0.4360	**Cell body**			
Input resistance (MΩ)	179.77 ± 11.22	188.70 ± 7.04	0.4890	x (µm)	1699.43 ± 19.61	1671.66 ± 16.54	0.2807
Membrane capacitance (pF)	147.90 ± 5.81	138.11 ± 4.05	0.1625	y (µm)	408.38 ± 6.00	412.52 ± 6.78	0.6559
τ_m_ (ms)	36.87 ± 1.71	35.30 ± 1.48	0.4881	Area (µm²)	126.43 ± 6.87	124.18 ± 3.97	0.7798
Sag ratio (%)	19.58 ± 1.49	19.68 ± 0.01	0.9559	**Basal dendrites**			
Current threshold (pA)	95.83 ± 4.58	93.79 ± 3.95	0.7360	Number	4.80 ± 0.33	3.90 ± 0.23	*0.0378
Voltage threshold (mV)	−41.33 ± 0.32	−41.95 ± 0.40	0.2486	Nodes	4.70 ± 1.01	3.10 ± 0.80	0.2296
AP amplitude (mV)	59.91 ± 0.45	59.35 ± 0.58	0.4672	Ends	9.60 ± 1.23	7.10 ± 0.81	0.1069
dv/dt_max_ (mV/ms)	526.34 ± 17.36	515.66 ± 15.16	0.6436	Total length (µm)	573.49 ± 89.81	476.44 ± 111.45	0.5064
dv/dt_min_ (mV/ms)	−61.84 ± 1.20	−61.29 ± 1.02	0.7276	Mean length (µm)	117.87 ± 13.30	129.35 ± 33.32	0.7526
AHP (mV)	−48.43 ± 0.38	−48.72 ± 0.47	0.6355	Complexity	2844.34 ± 799.60	2744.65 ± 1012.92	0.9393
AHP time to peak (ms)	4.61 ± 0.08	4.79 ± 0.11	0.1955	**Apical dendrite**			
50% AP width (ms)	1.21 ± 0.02	1.24 ± 0.02	0.2802	Nodes	10.10 ± 1.16	9.80 ± 1.81	0.8904
I-F slope (Hz/100pA)	15.83 ± 0.70	15.62 ± 0.49	0.7998	Ends	11.40 ± 1.11	11.00 ± 1.78	0.8510
1^st^ AP latency (ms)	142.67 ± 10.18	134.66 ± 8.85	0.5539	Total length (µm)	1093.56 ± 139.81	1119.48 ± 165.38	0.9060
Average ISI (ms)	49.84 ± 0.40	49.32 ± 0.40	0.3761	Complexity	91471.94 ± 28581.70	102676.71 ± 34763.74	0.8062
Ratio ISI_1_/ISI_n_	0.34 ± 0.02	0.32 ± 0.02	0.3738				

Values are mean ± SEM, with sample size in parenthesis. P-values were determined by Student’s t test. x, y cell coordinates relative to the dorsal apex and midline of the coronal slice respectively, RMP resting membrane potential, τ_m_ membrane time constant, AP action potential, AHP after-hyperpolarization, dv/dt_max_ peak depolarization velocity, dv/dt_min_ min peak repolarization velocity, ISI interspike interval, I-F input-frequency; *p < 0.05.

Correspondingly, the morphology of the apical and basal dendrites of L5 cells was similar between SNI and sham treated mice (Table 4). Sholl analysis, likewise, showed similar numbers of intersections (2-way ANOVA, p > 0.05) and branching (2-way ANOVA, p > 0.05) in both apical and basal dendrites of L5 pyramidal neurons of the two groups (Fig. 4f,g).

## Discussion

In the current study, we used a combined electrophysiological and morphological approach to assess alterations of mPFC pyramidal neurons induced by neuropathic nerve injury with a special focus on subregional and laminar specificity. We report a differential modulation of neuronal excitability in L2/3 and L5 of the PrL following SNI. In contrast, IL pyramidal cells were functionally unaltered in all layers investigated. Anatomical reconstruction of the recorded neurons showed that neuropathic pain was associated with increased dendritic length and complexity of pyramidal cells in L2/3 of the IL. Limited structural rearrangements were also revealed in L2/3 and L5 neurons of the PrL that were, however, only detected by more detailed analysis of their morphological characteristics.

Our results build on previous studies showing that stimulation of deeper layers of the mPFC alleviates both the sensory and affective components of pain and that their inhibition worsens nocifensive and motivational behaviour^13,43–47^. Specifically, we found that PrL L2/3 pyramidal neurons showed an increased input resistance and a more depolarized resting membrane potential 7 days after SNI treatment, indicative of increased excitability. These findings are in line with Cordeiro Matos, *et al*.^32^ reporting higher input resistance and neuronal activity in superficial layers of the mPFC of SNI treated rats three weeks after surgery. Persistent pain in SNI treated rats 7 days after surgery is associated with increased synaptic signalling in L2/3 pyramidal neurons due to an increase in the NMDA/AMPA ratio^15^. Moreover, overexpression of the NMDA receptor subunit NR2B in the mPFC increases responsiveness to inflammatory stimuli^48^.

However, SNI induced effects on passive and active membrane properties of L2/3 pyramidal cells are controversial and this may be related to pooling neuronal recordings from different neighbouring regions that show small but important differences, such as more dorsal regions corresponding to ACC rather than PrL^15,30,32^.

In contrast to pyramidal cells in the superficial PrL cortex, deep PrL L5 pyramidal neurons of SNI mice generated significantly lower firing rates in response to suprathreshold depolarizing current injections compared to controls. This is in line with previous studies reporting reduced excitability and action potential discharge activity of L5 PrL neurons within the mPFC in the SNI model of neuropathic pain^42,43^. A possible explanation for functional deactivation of the PrL cortex in neuropathic pain could be a reduction in glutamatergic currents in L5 pyramidal neurons of the mPFC of SNI rats^30^. More likely, local or amygdala driven feed-forward inhibition may account for neuron deactivation as documented in rat models of arthritis and SNI^43^. These *in vitro* data are corroborated by an *in vivo* study demonstrating reduced basal spontaneous as well as pain-evoked activity in the PrL in a rat model of persistent inflammatory pain^49^.

We also observed important differential effects of SNI on pyramidal cell excitability between the PrL and IL cortical regions (Supplemental Table 2). This could be explained by differential inputs that PrL and IL cortices receive from subcortical areas, in particular the basolateral amygdala^50^, which is reflected by the different roles of these areas in fear learning and memory^51–53^. Previous studies mainly focused on the ACC and the PrL cortices, since experimental lesions within these areas reduced mechanical hypersensitivity as well as conditioned-place aversion, whereas lesions of the IL cortex did not^54,55^.

So far, electrophysiological studies investigating the role of IL cortex in pain processing have not been conclusive. We demonstrate that functional properties of pyramidal cells in both superficial and deep cortical layers in the IL cortex are unaltered in neuropathic mice, which is in line with Cheriyan and Sheets^42^. Other studies, however, report changes in the IL, including loss of parvalbumin expressing (PV^+^) neurons and reduction of axon initial segment length in L5/6 neurons of the IL but not PrL three weeks after SNI injury^56^.

Apart from functional changes, pyramidal cells in superficial layers of the mPFC were shown to undergo morphological structural changes following SNI^15^. Our findings are consistent with this report, also demonstrating increased dendritic branching only in the basal but not the apical dendrites of PrL L2/3 pyramidal cells in SNI mice. In addition, the apical and basal dendrites of L5 PrL pyramidal cells showed a moderately increased branching after SNI, but no significant changes in total dendritic length and complexity. These findings appear at odds with a recent study reporting reduced length and branching of apical dendrites, along with reduced glutamatergic currents, in the mPFC of SNI rats^30^. Kelly, *et al*.^30^ also reported an increased input resistance and a reduced membrane capacitance of these cells that we could not observe in our experiments. Although species-specific differences cannot be ruled out, the reasons for this discrepancy are at present unclear and warrant further investigations.

Pyramidal neurons in deeper cortical layers of the IL were morphologically unaltered by SNI 7 days after surgery. This supports our electrophysiological data, as we observed no difference in the excitability of the recorded neurons in this layer. However, superficial pyramidal cells of the IL showed increased dendritic branching at both basal and the apical dendrites. With L2/3 representing the main mPFC input region, this increased dendritic complexity could represent a compensatory mechanism in response to a reduced glutamatergic input from the ventral hippocampus and mediodorsal thalamus^57^. That neuropathic pain leads to changes in dendritic branching correlates well with the upregulation of genes important for axonal guidance and maturation of dendritic spines, observed in the mPFC of SNI mice^58^.

The reasons for subregion- and layer-specific changes can be manifold. One possibility may be alterations in the activity of different local inhibitory circuits. Different GABAergic interneuron types differ in how they synapse on projection neurons, and thereby in how they control excitability and action potential integration before an output is generated^59^. They are distributed in a specific manner across different cortical layers^60^. For example, somatostatin (SOM^+^) and PV^+^ interneurons in L5 of the somatosensory cortex show reduced activity in the SNI mouse model, whereas vasointestinal polypeptide-expressing (VIP^+^) interneurons exhibit increased activity^61^. Consistently, the activity of PV^+^ interneurons drives the increase in the inhibitory GABAergic tone in PrL L5 of SNI operated mice^43^, while the excitation profile of PrL L2/3 PV^+^ interneurons and SOM^+^ interneurons across both layers is unaltered in the chronic constriction injury mouse model^42^.

Alternatively, altered input to the mPFC may be sufficient to explain the subregion- and layer-specific differences. Terminals from the ventral hippocampus are distributed unevenly in the mPFC, with more terminals ending in ventral regions^57^, and the basolateral amygdala (BLA) projecting to layer 2 neurons of the PrL rather than the IL^50^. Interestingly, BLA projections preferentially target neurons projecting to the periaquaeductal grey, the main descending pain control hub^50^. This top-down control of the descending pain-pathway has also been implicated as a possible target contributing to the chronification of pain, with facilitation of either direct ACC-to-spinal cord^62^ or sensory cortex-to-spinal cord^63^ projections. Therefore, modulation of specific types of interneurons limited to specific mPFC subregions and layer specific projections from relevant pain processing brain regions could be responsible for the currently observed differences.

Also, it should be noted that the changed electrophysiological and morphological properties observed in the different layers and subregions of the mPFC might vary between different timepoints after nerve injury, and might therefore be associated with different stages of neuropathy and pain chronification.

Studies on humans and rodents strongly suggest that males and females show different sensitivity to pain and differences in peripheral or spinal processing of painful stimuli^64,65^. Women show stronger activation of the mPFC than men in response to subthreshold and strong painful stimuli, which could be linked to increased self-related attention in response to pain^66^ and only female mice show differential pERK activation in mPFC in a partial nerve ligation model^67^. In the current investigation all recordings were performed in male mice since male SNI mice are significantly more impaired in a set-shifting task for prefrontal cortex dysfunction than female mice^56^. However, additional studies are required to obtain mechanistic insight into sex-specific circuits for the processing of nociceptive stimuli in the mPFC.

In the present study we provide the first systematic investigation of subregion and layer specific neuronal changes occurring in the PrL and IL cortices of neuropathic mice, thereby providing new insights into mPFC neuronal reorganization associated with the chronification of pain.

In conclusion, our results support the view that neuropathic pain results from region- and layer-specific functional as well as structural changes of the mPFC. The dissection of these complex alterations may contribute to a better understanding of the widespread plasticity events occurring in different brain regions as a consequence of long-lasting pain, and possibly to the identification of novel strategies for the development of more effective, mechanism-targeted treatments.

## Supplementary information

Supplemental Figures and Tables

## Data Availability

The datasets generated during and/or analyzed during the current study are available from the corresponding author on reasonable request.
