# Supplementary material for: Layer- and subregion-specific electrophysiological and morphological changes of the medial prefrontal cortex in a mouse model of neuropathic pain
Source: Sci Rep. 2019 Jul 1;9:9479. doi: 10.1038/s41598-019-45677-z (PMC6603192; doi:10.1038/s41598-019-45677-z)
Supplement: Supplementary file 1 — Supplemental Figures and Tables [file 41598_2019_45677_MOESM1_ESM.pdf]

Layer- and subregion-specific electrophysiological and morphological changes of the medial prefrontal cortex in a mouse model of neuropathic pain

Miodrag Mitrić[1], Anna Seewald[2], Giorgia Moschetti[3], Paola Sacerdote[3], Francesco Ferraguti[2], Kai K. Kummer[1*] and Michaela Kress[1]

[1] Division of Physiology, Medical University of Innsbruck, Innsbruck, Austria

[2] Department of Pharmacology, Medical University of Innsbruck, Innsbruck, Austria

[3] Department of Pharmacological and Biomolecular Sciences, University of Milan, Milan, Italy

[*] **Corresponding author**

Kai Kummer, PhD
Division of Physiology
Medical University of Innsbruck
Schoepfstrasse 41/EG
6020 Innsbruck
Austria

Phone: 0043-650-9700514
Fax: 0043-512-9003-73800
E-Mail: kai.k.kummer@gmail.com

Running Title: *Excitability of medial prefrontal cortex in neuropathic pain*

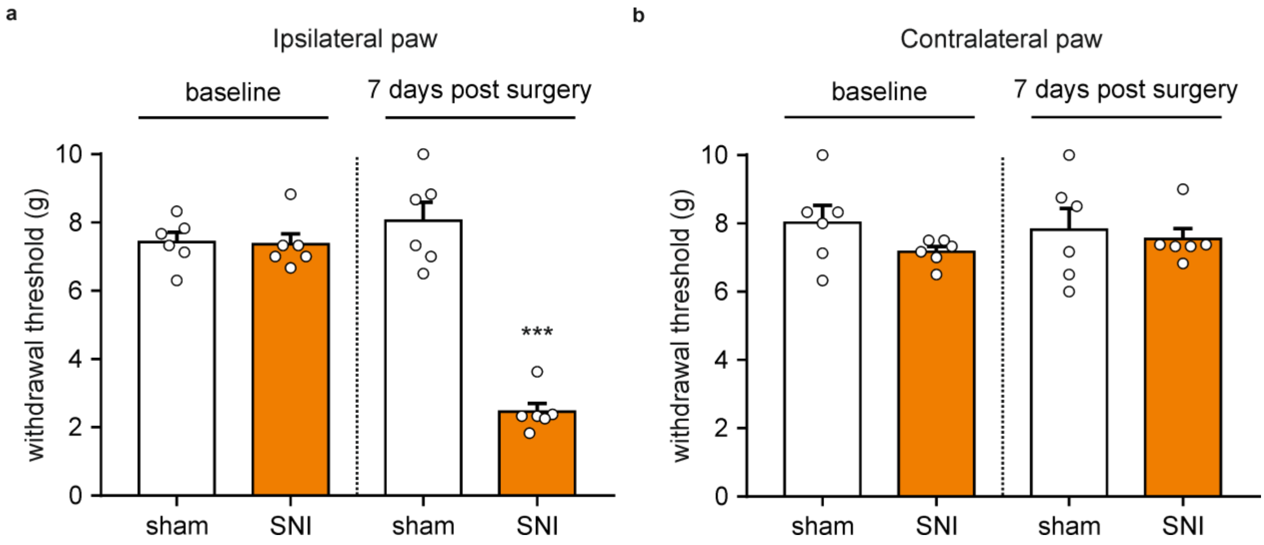

Supplemental Figure 1. Development of mechanical allodynia in the operated paw 7 days after SNI. Mechanical threshold measured using the dynamic plantar aesthesiometer at baseline and 7 days after SNI or sham surgery. (a) Mechanical threshold was significantly lower in the ipsilateral hindpaw of SNI mice (2-way ANOVA with repeated measures, $p<0.001$). (b) Mechanical threshold of the contralateral paw was unaffected by the SNI surgery (2-way ANOVA with repeated measures, $p>0.05$).

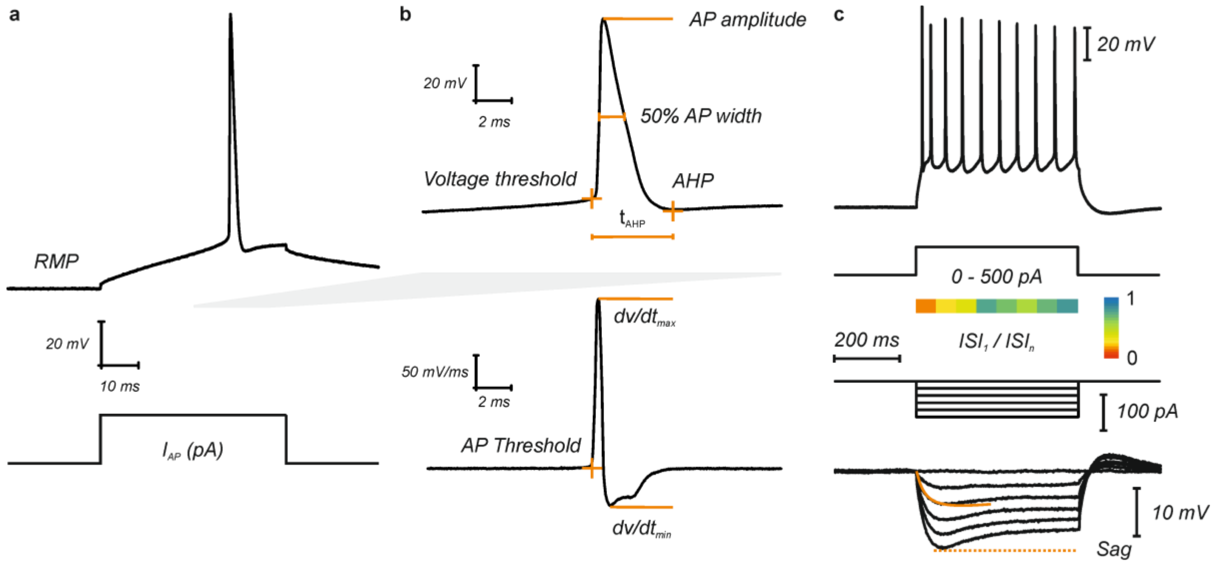

Supplemental Figure 2. Passive and active membrane properties of recorded neurons. (a) AP elicited by a 50 ms depolarizing current pulse. (b) AP analysis (top), AP first derivative (bottom). (c) AP firing pattern of a recorded neuron elicited in response to a 500 ms depolarizing current step. Below, adaptation ratios from 1st to the n-1st interspike interval relative to the last (n^{th}) presented as a heat map. Bottom, response to hyperpolarizing current injections to calculate input resistance and voltage sag ratio.

Investigated biophysical parameters from whole-cell patch-clamp recordings

**RMP (mV)**	Averaged 1-minute voltage value in the absence of DC injection.	
**Input resistance (MΩ)**	Calculated from the linear fit of the voltage-current response to 20 pA hyperpolarizing current injection steps from -100 to 0 pA.	
**Membrane capacitance (pF)**	Membrane time constant obtained by a double exponential fit of the voltage response to a −40 pA current injection (Supplemental Figure 2c).	
**τ_m (ms)**		
**Sag ratio (%)**	$(V_{ss}-V_{min})$ / $(V_{min}$-RMP)	mPFC: V_{min}-RMP ~ -7.5 mV
**Current threshold, I_{AP} (pA)**	Minimum 50 ms depolarizing current pulse needed to induce an AP	
**Voltage threshold (mV)**	Voltage at the time point when the depolarization speed exceeded 10 mV/ms.	
**AP amplitude (mV)**	Voltage amplitude at the peak of the AP.	
**dv/dt_{max} (mV/ms)**	Maximum speed of depolarization.	
**dv/dt_{min} (mV/ms)**	Minimum speed of repolarization.	
**AHP (mV)**	Voltage difference between the first action potential threshold and the most negative voltage associated with the slower AHP following that action potential.	
**AHP time to peak (ms)**	Time from AP threshold to AHP.	
**50% AP width (ms)**	AP duration at half-maximal amplitude of the first action potential.	
**I-F slope (Hz/100pA)**	Linear slope of the polynomial fit of the AP frequency relative to 500 ms injected depolarized current steps (I-F) x 100.	
**1st AP latency (ms)**	The time between the onset of a 500 ms depolarizing current and the threshold of the first action potential.	
**Average ISI (ms)**	Averaged interspike intervals.	trace with at least 10 APs
**Ratio ISI_1/ISI_n**	Ratio of the first and the last interspike interval.	
**Access resistance (Rs)**	Calculated from capacitive transients in response to 10 ms square wave depolarizing pulses according to the Ohm's law.	

Supplemental Table 1. Electrophysiological parameters. RMP resting membrane potential, AP action potential, AHP afterhyperpolarization, ISI interspike interval, I-F input-frequency, V_{ss} voltage at steady state, V_{min} minimum value reached after the beginning of the current injection.

**Electrophysiology**	**L2/3 PrL (n=28)**	**L2/3 IL (n=15)**	**P value**	**L5 PrL (n=29)**	**L5 IL (n=24)**	**P value**
**x (µm)**	917.64 ± 29.51	1679.26 ± 25.04	*0.0000	933.61 ± 25.97	1699.43 ± 19.61	*0.0000
**y (µm)**	222.14 ± 5.37	212.53 ± 5.50	0.2582	427.55 ± 6.98	408.38 ± 6.00	*0.0470
**RMP (mV)**	-77.08 ± 0.81	-72.33 ± 1.44	*0.0033	-67.29 ± 0.45	-66.31 ± 0.65	0.2123
**Input resistance (MΩ)**	140.96 ± 10.51	241.38 ± 15.16	*0.0000	167.62 ± 13.76	179.77 ± 11.22	0.5088
**Membrane capacitance (pF)**	171.89 ± 10.41	123.00 ± 5.10	*0.0015	193.56 ± 5.82	147.90 ± 5.81	*0.0000
**τ_m (ms)**	22.33 ± 1.59	42.79 ± 4.01	*0.0000	38.59 ± 2.71	36.87 ± 1.71	0.6113
**Sag ratio (%)**	4.55 ± 0.26	3.92 ± 1.51	0.5957	16.47 ± 1.63	19.58 ± 1.49	0.1727
**Current threshold (pA)**	214.29 ± 16.50	126.88 ± 9.95	*0.0005	114.83 ± 5.41	95.83 ± 4.58	*0.0117
**Voltage threshold (mV)**	-39.06 ± 0.38	-37.59 ± 0.61	*0.0370	-43.50 ± 0.52	-41.33 ± 0.32	*0.0015
**AP amplitude (mV)**	57.14 ± 0.54	57.32 ± 0.71	0.8470	57.84 ± 0.60	59.91 ± 0.45	*0.0103
**dv/dt_{max} (mV/ms)**	430.38 ± 19.01	389.33 ± 17.72	0.1579	467.14 ± 16.17	526.34 ± 17.36	*0.0161
**dv/dt_{min} (mV/ms)**	-56.38 ± 1.87	-58.53 ± 1.98	0.4610	-63.21 ± 1.21	-61.84 ± 1.20	0.4308
**AHP (mV)**	-46.89 ± 0.51	-48.28 ± 0.60	0.0952	-48.88 ± 0.47	-48.43 ± 0.38	0.4737
**AHP time to peak (ms)**	4.99 ± 0.14	5.12 ± 0.13	0.5560	5.04 ± 0.22	4.61 ± 0.08	0.0961
**50% AP width (ms)**	1.33 ± 0.04	1.28 ± 0.03	0.4161	1.22 ± 0.02	1.21 ± 0.02	0.7911
**I-F slope (Hz/100pA)**	12.45 ± 0.53	15.58 ± 0.66	*0.0007	14.69 ± 0.35	15.83 ± 0.70	0.1275
**1st AP latency (ms)**	167.99 ± 8.30	223.30 ± 25.92	*0.0174	160.47 ± 11.41	142.67 ± 10.18	0.2589
**Av ISI (ms)**	51.11 ± 1.20	50.46 ± 0.44	0.6556	50.37 ± 0.25	49.84 ± 0.40	0.2431
**Ratio ISI_1/ISI_n**	0.31 ± 0.02	0.37 ± 0.02	0.0586	0.39 ± 0.03	0.34 ± 0.02	0.1789

Supplemental Table 2. Comparison between prelimbic and infralimbic regions in sham mice. Values are mean ± SEM, with sample size in parenthesis. P-values were determined by Student's t test. x, y cell coordinates relative to the dorsal apex and midline of the coronal slice respectively, RMP resting membrane potential, τ_m membrane time constant, AP action potential, AHP after-hyperpolarization, dv/dt_{max} peak depolarization velocity, dv/dt_{min} min peak repolarization velocity, ISI interspike interval, I-F input-frequency; * $p<0.05$.
